# Detection of Active Matriptase Using a Biotinylated Chloromethyl Ketone Peptide

**DOI:** 10.1371/journal.pone.0077146

**Published:** 2013-10-18

**Authors:** Sine Godiksen, Christoffer Soendergaard, Stine Friis, Jan K. Jensen, Jette Bornholdt, Katiuchia Uzzun Sales, Mingdong Huang, Thomas H. Bugge, Lotte K. Vogel

**Affiliations:** 1 Department of Cellular and Molecular Medicine, University of Copenhagen, Copenhagen, Denmark; 2 Department of Biology, University of Copenhagen, Copenhagen, Denmark; 3 Proteases and Tissue Remodeling Unit, National Institute of Dental and Craniofacial Research, Bethesda, Maryland, United States of America; 4 Department of Molecular Biology and Genetics, Aarhus University, Aarhus, Denmark; 5 Danish-Chinese Centre for Proteases and Cancer; 6 State Key Lab of Structural Chemistry, Fujian Institute of Research on the Structure of Matter, Fuzhou, Fujian, China; Stanford University, United States of America

## Abstract

Matriptase is a member of the family of type II transmembrane serine proteases that is essential for development and maintenance of several epithelial tissues. Matriptase is synthesized as a single-chain zymogen precursor that is processed into a two-chain disulfide-linked form dependent on its own catalytic activity leading to the hypothesis that matriptase functions at the pinnacle of several protease induced signal cascades. Matriptase is usually found in either its zymogen form or in a complex with its cognate inhibitor hepatocyte growth factor activator inhibitor 1 (HAI-1), whereas the active non-inhibited form has been difficult to detect. In this study, we have developed an assay to detect enzymatically active non-inhibitor-complexed matriptase by using a biotinylated peptide substrate-based chloromethyl ketone (CMK) inhibitor. Covalently CMK peptide-bound matriptase is detected by streptavidin pull-down and subsequent analysis by Western blotting. This study presents a novel assay for detection of enzymatically active matriptase in living human and murine cells. The assay can be applied to a variety of cell systems and species.

## Introduction

Matriptase (also known as MT-SP1, epithin, TADG-15 and SNC19) is a type II transmembrane serine protease that is expressed in most epithelia and has pleiotropic roles in epithelial development and homeostasis [1]–[5]. Matriptase is a multimodular, approximately 95 kDa protease that consists of a short cytoplasmic N-terminal peptide, a signal anchor that functions as a single-pass transmembrane domain, a sea urchin sperm protein, enteropeptidase, agrin (SEA) domain, two complement C1r/s urchin embryonic growth factor and bone morphogenetic protein-1 (CUB) domains, four low-density lipoprotein receptor class A (LDLRA) domains, and a trypsin-like serine protease domain (SPD) [6], [7].

Matriptase, like other trypsin-like serine proteases, is synthesized as a catalytically inactive, single chain protein (zymogen). The activation of matriptase zymogen (also termed zymogen conversion) is extraordinarily complex and still incompletely understood at the mechanistic level. Matriptase activation involves two sequential endoproteolytic cleavages, and may also require transient interactions with its cognate inhibitor, hepatocyte growth factor activator inhibitor (HAI)-1 [7], [8] [reviewed in [9], [10]] or HAI-2 [11]. Full-length matriptase is first hydrolysed at the Gly149-Ser150 peptide bond, which is located in a conserved GSVIA motif within the SEA domain, whereby the SEA domain-cleaved zymogen form is generated. The protease remains attached to the membrane by strong non-covalent interactions within the cleaved SEA domain. The SEA domain cleavage appears to occur within the secretory pathway, as only the SEA domain-cleaved form of the protease is present on the surface of cells [12]. Matriptase is next converted into its active conformation by proteolytic cleavage after Arg614 within the conserved activation cleavage site R-VVGG located within the serine protease domain. Importantly, this cleavage has been reported to require the proteolytic activity of matriptase, as mutations in any of the residues of the catalytic triad renders matriptase unable to undergo activation site cleavage. This finding has led to a model for matriptase activation in which a weak intrinsic proteolytic activity of the SEA domain-cleaved matriptase zymogen activates neighboring SEA domain-cleaved matriptase molecules [7]. Consistent with this model, the purified SEA domain-cleaved soluble matriptase has been shown to be capable of hydrolyzing synthetic peptide substrates in solution, although catalytic activity of the cell surface matriptase zymogen still needs to be demonstrated [13], [14].

The capacity of matriptase zymogen to autoactivate is unusual and has led to the proposal that matriptase serves as an initiator of proteolytic cascades. Indeed, matriptase has been shown to facilitate activation of the urokinase plasminogen activation cascade, of epidermal kallikreins, and of the GPI-anchored serine protease, prostasin [15]–[17]. Recent studies, however, have unexpectedly shown that matriptase activation in some contexts is critically dependent on prostasin [18]–[20]. This suggests that prostasin may directly mediate the activation site cleavage of matriptase, that matriptase activates an unidentified ternary matriptase-activating protease or that prostasin serves as a non-enzymatic allosteric co-factor for matriptase autoactivation. The specific physiological mechanisms that trigger the complex series of events leading to the activation of matriptase are also poorly understood. In cultured cells, matriptase activation has been reported to occur in response to exposure to sphingosine-1-phosphate, suramin, androgens, low pH, and either soluble or membrane-anchored prostasin [19], [21]–[27].

The absence of probes to specifically detect active non-inhibitor complexed matriptase *in situ* has been a principal obstacle in unraveling the complex biochemistry of the protease. In this paper, we have combined antibody specificity with the high affinity of biotin-streptavidin interaction to design a peptide inhibitor-based assay for the detection of matriptase activity. Specifically, we have engineered a chloromethyl ketone-based tetra-peptide with an N-terminal biotin moiety that allows for the segregation of non-reacting proteins, including forms of matriptase that are not able to bind the peptide. Specificity of the assay is obtained by Western blot analysis employing specific antibodies against matriptase. We validate the probe in human and murine cell-based models, show that active matriptase represent only a minor fraction of total cell surface matriptase, and demonstrate that cell surface matriptase zymogen is catalytically active.

## Materials and Methods

### Chromogenic assay

Matriptase SPD expressed and purified from *P. pastoris* [28] was prepared in 20 mM Hepes pH 7.4, 140 mM NaCl supplemented with 0.1% BSA (Sigma-Aldrich, Copenhagen, Denmark) and 100 µl was added to wells of a 96 well plate at a concentration of 0.4 nM. Stocks of varying concentrations (5 nM or 50 µM) of biotin-Arg-Gln-Arg-Arg-CMK (biotin-RQRR-CMK) peptide (American Peptide, Derbyshire, UK) were incubated in the same buffer at 37°C for up to 3 h. At specific time points, 100 µl of inhibitor solution was added to the well containing the active catalytic domain of matriptase. Following additional 10 min incubation at 37°C, 10 µl 6.3 mM chromogenic substrate H-D-Isoleucil-L-prolyl-L-arginine-p-nitroaniline (cat. no. S2288, Chromogenix, Essen, Germany) was added and substrate conversion was followed in a standard plate reader by 30 min continuous measurements of the absorbance at 405 nm at 37°C. The rate of substrate turnover was determined from the slope of the corresponding curve of color development resulting from a pseudo first order reaction. To test pH effects on substrate turnover and biotin-RQRR-biotin binding efficiency, the buffer component of the assay was changed from Hepes, pH 7.4 to 20 mM Citric acid pH 6.0.

### Cell culture

The human colon epithelial cell line Caco-2 [29] was grown in Dulbecco's modified Eagle medium supplemented with 2 mM L-glutamine, 20% fetal bovine serum (Gibco, Copenhagen, Denmark), 1× non-essential amino acids, 100 units/ml penicillin and 100 µg/ml streptomycin (Invitrogen, Copenhagen, Denmark) at 37°C in an atmosphere of 5% CO_2_. For all experiments, 1–2×10^6^ cells were seeded into 35 mm tissue culture plates or 0.4 µm-pore-size 24 mm Transwell® filters (Corning, Copenhagen, Denmark) allowing separate access to the apical and the basolateral plasma membrane. The cell culture medium was changed every day. Filter-grown cells were cultivated until 11 days post-confluence before they were used in experiments. The tightness of filter-grown cells was assayed by filling the inner chamber to the brim and allowing it to equilibrate overnight.

### Ethics Statement

All animal work was performed in accordance with protocols approved by the National Institute of Dental and Craniofacial Research Animal Care and Use Committee (Animal Study Proposal Number: 10-577).

### Isolation and short-term culture of primary keratinocytes from newborn mice

Epidermis was isolated from newborn mice (p1-2) and grown in culture as previous described [30]. Briefly, newborn pups were euthanized by decapitation and the torso was submerged in betadine and ethanol to sterilize the skin. The skin was incubated in 0.25% trypsin w/o EDTA (Sigma-Aldrich) o/N at 4°C. The dermal portion was discarded, and the epidermis was minced to release keratinocytes. The minced epidermis was resuspended in 45 µM Ca^2+^/10% FBS/Keratinocyte-SFM (Invitrogen) media and was filtered through a 100 µm cell strainer and centrifuged to remove stratum corneum pieces. The cell pellet was resuspended in low calcium medium (45 µM Ca^2+^/Keratinocyte-SFM media) and plated in culture plates coated with collagen I (BD Biosciences, New Jersey, USA). Cells were grown in low calcium medium to sub-confluence and cell culture medium was changed every second day.

### Labeling with biotin-Arg-Gln-Arg-Arg-chloromethyl ketone (biotin-RQRR-CMK) peptide inhibitor and S-NHS-SS-biotin

Cells were washed twice; filter-grown Caco-2 cells with PBS^++^ (PBS supplemented with 0.7 mM CaCl_2_ and 0.25 mM MgCl_2_) and primary murine keratinocytes with PBS. For labeling of active matriptase, cells were incubated with 50 µM biotin-RQRR-CMK (American Peptide) in serum-free minimal essential medium (MEM) eagle with Earle's supplemented with 0.2% NaHCO_3_, 100 units/ml penicillin and 100 µg/ml streptomycin (Invitrogen) at 37°C for the times indicated. As a negative control, cells were treated with 50 µM of a corresponding peptide without a CMK group; biotin-Arg-Gln-Arg-Arg (biotin-RQRR). Peptides were prepared as 50 mM stocks in DMSO and were stored at −20°C. For acid-induced activation of matriptase, cells were labeled in a physiological phosphate buffer (25 mM Na_2_HPO_4_, 175 mM NaH_2_PO_4_) pH 6.0 or pre-treated with physiological phosphate buffer pH 6.0 before labeling in serum-free MEM eagle with Earle's supplemented with 0.2% NaHCO_3_, 100 units/ml penicillin and 100 µg/ml streptomycin. For labeling of surface proteins, cells were biotinylated from the basolateral side with 1 mg/ml EZ-link™ Sulfo-NHS-SS-Biotin (Pierce) dissolved in PBS^++^ for 30 min at 4°C as previously described [31]. After peptide- and/or ordinary biotin-labeling, the cells were washed four times with ice-cold PBS^++^. For biotin-labeling, residual biotin was quenched with 50 mM glycine/PBS^++^ for 5 min at 4°C and the cells were washed twice with PBS^++^. Cells were lysed in PBS containing 1% Triton X-100, 0.5% deoxycholate and protease inhibitors (10 mg/l benzamidine, 2 mg/l pepstatin A, 2 mg/l leupeptin, 2 mg/l antipain, and 2 mg/l chymostatin). Insoluble material was precipitated at 20,000×*g* for 20 min at 4°C and equal amounts of supernatants were transferred to clean Eppendorf tubes.

### Streptavidin pull down

Cleared lysates were incubated for 2 h with end-over-end rotation at 4°C with 50 µl/24 mm filter pre-washed streptavidin-coated resin (Pierce), prepared as described by manufacturer. The streptavidin-coated resin was washed four times with 25 mM TRIS-HCl, 500 mM NaCl, 0.5% Triton X-100, pH 7.8, and three times with 10 mM TRIS-HCl, 150 mM NaCl, pH 7.8. Biotinylated proteins were eluted from the streptavidin-coated resin by boiling in SDS sample buffer.

### SDS-PAGE and Western blotting

Proteins were separated on 10% acrylamide gels and transferred to Immobilon-P PVDF membranes (Millipore, Copenhagen, Denmark). The membranes were blocked with 10% non-fat dry milk in PBS containing 0.1% Tween-20 (PBS-T) for 1 hr at RT and were probed with primary antibody diluted in 1% non-fat dry milk in PBS-T at 4°C o/N. The next day the membranes were washed 3 times with PBS-T, followed by detection of bound primary antibody with horseradish peroxidase (HRP)-conjugated secondary antibody (Pierce) or alkaline phosphatase (AP)-conjugated secondary antibody (Sigma-Aldrich). After 3 washes with PBS-T, the signal was developed using the ECL reagent Super Signal West Femto Maximum Sensitivity Substrate (Pierce) for HRP-conjugated secondary antibodies according to the protocol supplied by the manufacturer and visualized with a Fuji LAS1000 camera (Fujifilm, Stockholm, Sweden) or by nitro-blue tetrazolium and 5- bromo-4-chloro-3′-indolyphosphate (Pierce) according to the protocol supplied by the manufacturer for AP-conjugated secondary antibody.

### Antibodies

The antibodies used for detection of matriptase in Western blotting were monoclonal mouse anti-human matriptase antibody M32 or monoclonal mouse anti-human matriptase antibody M24 (1 µg/ml 1% milk in PBS-T), which detects a 70 kDa band (representing both active and zymogen matriptase both in boiled and non-boiled samples) and the 120–130 kDa complex of matriptase with HAI-1, which can only be detected in non-boiled samples [32]; monoclonal mouse anti-human matriptase antibody M69 (1 µg/ml 1% milk in PBS-T), which recognizes the 120–130 kDa complex of matriptase with HAI-1 [32]; monoclonal mouse anti-human HAI-1 antibody M19 (1 µg/ml 1% milk in PBS-T), which recognizes free 55 kDa HAI-1 and the 120–130 kDa matriptase-HAI-1 complex [32]; polyclonal rabbit anti-human matriptase (1.5 µg/ml 1% milk in PBS-T) raised against the SPD of matriptase recognizing a 70 kDa band (both active and zymogen matriptase) under non-reducing conditions and the 70 kDa zymogen form and the 30 kDa serine protease domain of cleaved matriptase under reducing conditions (Cat. no. IM1014, Calbiochem). For detection of matriptase in murine cells, sheep anti-matriptase was used (AF3946 diluted 1∶1000, R&D). Secondary antibodies include goat anti-mouse HRP-conjugated (2 ng/ml 1% milk in PBS-T) (Pierce), goat anti-rabbit HRP-conjugated (1.5 µg/ml 1% milk in PBS-T) (Pierce) and donkey anti-sheep AP-conjugated (2 ng/ml 1% milk in PBS-T) (Sigma-Aldrich).

## Results

### Biotin-RQRR-CMK peptide inhibitor designed to react with active matriptase

**Figure 1:**
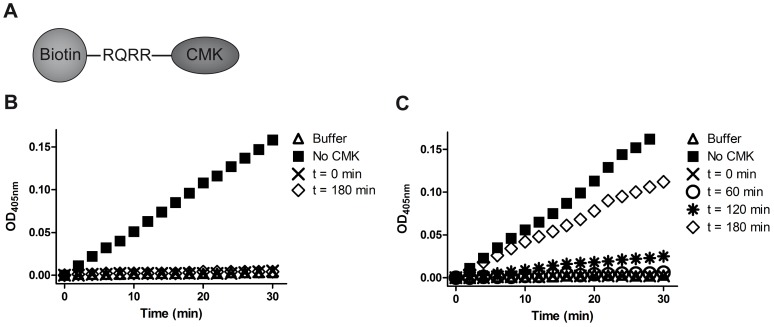
Biotin-RQRR-CMK efficiently inhibits matriptase even after 3 hours of pre-incubation at 37°C. (A) Schematic structure of the biotin-RQRR-CMK peptide inhibitor. (B) The reactivity of biotin-RQRR-CMK was tested after 180 min of pre-incubation at 37°C (diamonds) or without preincubation (crosses). 0.2 µM matriptase SPD was incubated for 10 min at 37°C with (diamonds and crosses) or without (squares) 50 µM biotin-RQRR-CMK before addition of the chromogenic substrate to a final concentration of 300 µM. (C) The stability of 5 nM biotin-RQRR-CMK was further tested after the time points 0 (crosses), 60 (circles), 120 (stars), and 180 min (diamonds) of pre-incubation at 37°C and compared to a control not containing biotin-RQRR-CMK (squares). As described above, 0.2 nM matriptase SPD was added to each sample and incubated for 10 min at 37°C followed by addition of the chromogenic substrate to a final concentration of 300 µM. In all cases, the enzymatic activity of SPD was monitored by conversion of the chromogenic substrate (S2288). Each plot shows the change in optical density at 405 nm of the reaction mixture as a function of reaction time. The presence of active protease results in a continued release of a yellow cleavage product resulting in a linear color development in agreement with a pseudo 1^st^ order reaction due to the high molar excess of substrate to protease. Results shown are representative of 3 independent experiments.

In order to detect active matriptase, we designed the peptide-based probe, biotin-RQRR-CMK, consisting of a tetra-peptide; RQRR with an N-terminal biotin moiety and a C-terminal CMK group (figure 1A). This inhibitor was designed based on a preferred substrate sequence of matriptase [33]. The CMK group ensures that the protease-peptide interaction results in the formation of a covalent bond between the peptide and the protease, thereby attaching a biotin moiety to the now inactivated enzyme [34]. In a complex media, the biotin group allows for efficient isolation of biotin-RQRR-CMK-labeled proteases and endogenously biotinylated proteins by streptavidin precipitation, whereby catalytically-inactive matriptase not able to bind the substrate is segregated away [35].

### Biotin-RQRR-CMK inhibits the proteolytic activity of matriptase SPD in vitro

To verify that biotin-RQRR-CMK binds and inhibits matriptase, we tested whether the peptide inhibitor was able to block the hydrolysis of a chromogenic substrate by recombinant matriptase SPD. Although matriptase has a pH optimum at pH 9 [33], this assay was performed at pH 7.4 in order to mimic the conditions on the plasma membrane. We found that 50 µM biotin-RQRR-CMK renders 0.2 nM matriptase SPD unable to cleave the chromogenic substrate (figure 1B, crosses), whereas matriptase SPD in the absence of biotin-RQRR-CMK cleaved the substrate efficiently (figure 1B, squares). Chloromethyl ketones are known to be hydrolyzed in aqueous solutions often with a half life 5–20 min [34]. To test the stability of the inhibitor, 50 µM biotin-RQRR-CMK was pre-incubated in aqueous solution at 37°C for 180 min before being added to the matriptase SPD. We found that the remaining active fraction of the pre-incubated biotin-RQRR-CMK is still sufficient to efficiently inhibit the activity of 0.2 nM matriptase SPD (figure 1B, diamonds). To further explore the stability of biotin-RQRR-CMK in aqueous solution, 0.2 nM matriptase SPD was incubated with just 5 nM biotin-RQRR-CMK after various times of pre-incubation at 37°C. Our experiments show that 5 nM biotin-RQRR-CMK is capable of fully inhibiting the peptidolytic activity of 0.2 nM matriptase SPD (figure 1C, crosses) even after 60 min of pre-incubation at 37°C (figure 1C, circles) whereas pre-incubation for 120 min or longer gradually reduces the efficiency of biotin-RQRR-CMK inhibition (figure 1C, stars and diamonds). This shows that biotin-RQRR-CMK is an efficient inhibitor of matriptase SPD and that biotin-RQRR-CMK is sufficiently stable in aqueous solutions to conduct the experiments described below. We have roughly estimated the half-life of biotin-RQRR-CMK to be 25 min under the experimental conditions described (data not shown).

### Biotin-RQRR-CMK reacts with a subset of matriptase molecules on the surface of cells in culture

Next, we addressed whether the biotin-RQRR-CMK peptide inhibitor is able to bind active matriptase on the surface of cells in culture. Differentiated Caco-2 cells express matriptase that can be detected mainly in the SEA domain-cleaved zymogen form but also in the Arg614-cleaved form in complex with HAI-1 [36], [37]. We have previously shown that activation site cleavage of matriptase after Arg614 takes place on the basolateral plasma membrane of 11 days post-confluent Caco-2 cells [36] indicating that these cells should at least momentarily contain Arg614-cleaved matriptase not in complex with HAI-1 on the basolateral plasma membrane if it exists. Therefore, in order to test whether biotin-RQRR-CMK is able to bind to plasma membrane-associated matriptase, we treated 11 days post-confluent filter-grown Caco-2 cells with biotin-RQRR-CMK from the basolateral side at 37°C for 2–180 min or at 4°C for 180 min. The inhibitor-treated cells were lysed, and biotin-RQRR-CMK-labeled proteases were extracted from cleared lysates using streptavidin-coated resin. Proteins were released from the resin by boiling in SDS sample buffer. Due to substrate overlap of matriptase with other trypsin-like serine proteases [33], we detected active matriptase by Western blot analysis using a matriptase specific antibody. To assess the steady state level of total surface-associated matriptase, parallel cultures were surface-biotinylated with S-NHS-SS-biotin at 4°C, whereby protein transport is inhibited. S-NHS-SS-biotin is plasma membrane impermeable and reacts with amine groups in an unspecific manner and therefore in this setup labels membrane-bound proteins in general.

**Figure 2:**
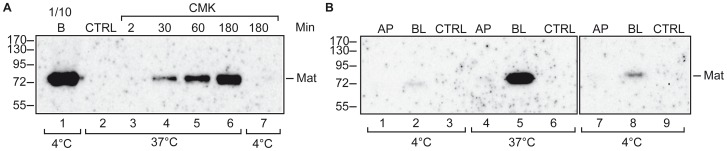
Biotin-RRQR-CMK reacts with a subset of matriptase molecules on the surface of Caco-2 cells. (A) Eleven days post-confluent Caco-2 cells grown on Transwell filters were labeled with 50 µM biotin-RQRR-CMK from the basolateral side for the times indicated (2–180 min) at 37°C (lanes 3–6) or for 180 min at 4°C (lane 7). As a measure of the steady state level of matriptase, membrane proteins on the basolateral plasma membrane of filter-grown Caco-2 cells were labeled by incubation with S-NHS-SS-biotin at 4°C for 30 min (lane 1). As a negative control, cells were labeled from the basolateral side with 50 µM control peptide; biotin-RQRR (lane 2). All cells were lysed and biotinylated proteins were precipitated using streptavidin-coated resin and were analyzed by non-reducing SDS-PAGE and Western blotting using the monoclonal matriptase antibody; M32. A tenth of the surface biotinylated sample was loaded (lane 1); whereas total sample volume was loaded for the other samples (lanes 2-7). (B) Caco-2 cells grown on Transwell filters were labeled with the biotin-RQRR-CMK peptide inhibitor from either the apical (lanes 1, 4, and 7) or the basolateral (lanes 2, 5, and 8) side for 180 min at either 4°C or 37°C. An overexposure of lanes 1–3 is shown in lanes 7–9. As a negative control, cells were labeled from the basolateral side with a peptide corresponding to the inhibitory peptide but lacking the CMK moiety (CTRL, lanes 3, 6, and 9). Cells were lysed and the lysates of multiple filters were pooled. Biotinylated proteins were precipitated using streptavidin-coated resin and the streptavidin pull downs were released by boiling in SDS-PAGE samples buffer and analyzed by SDS-PAGE and Western blotting using the monoclonal M32 antibody. Positions of the molecular weight markers (kDa) are indicated on the left. Results shown are representative of 3 independent experiments.

Matriptase endogenously expressed by Caco-2 cells binds biotin-RQRR-CMK, as demonstrated by Western blotting (figure 2A, lanes 4–6), and no matriptase could be detected when labeling with a control peptide; biotin-RQRR (figure 2A, lane 2: CTRL). This shows that matriptase is not endogenously biotinylated. Biotin-RQRR-CMK labeling displayed time and temperature dependence. The amount of matriptase reacting with biotin-RQRR-CMK within a 2 min labeling time frame was below the detection limit of the assay (figure 2A, lane 3). Likewise, labeling with biotin-RQRR-CMK at 4°C produced no significant signal (figure 2A, lane 7). Comparison of steady state surface-associated matriptase as determined by biotinylation with S-NHS-SS-biotin to the accumulated biotin-RQRR-CMK labeled matriptase over a 30 min period or longer shows that only a fraction of surface-associated matriptase on Caco-2 cells is able to bind biotin-RQRR-CMK (figure 2A, compare lane 1 to lanes 4–6). In the experiments described above, we are not able to distinguish whether the biotin-RQRR-CMK-reactive matriptase is present on the cell surface or whether biotin-RQRR-CMK is endocytosed and encounters matriptase in an intracellular compartment, as labeling was performed at 37°C. To verify that the biotin-RQRR-CMK-reactive matriptase is present on the plasma membrane, we performed a similar experiment, labeling 11 days post-confluent filter-grown Caco-2 cells from either the apical or the basolateral side for 180 min at 4°C or 37°C in triplicates and pooled the resulting cell lysates. This experiment showed that biotin-RQRR-CMK reactive matriptase is present on the basolateral plasma membrane at low levels, whereas reactive matriptase could not be detected on the apical plasma membrane of Caco-2 cells (figure 2B). Together this shows that only a fraction of the membrane-associated matriptase on the basolateral plasma membrane of Caco-2 cells binds biotin-RQRR-CMK. Biotin-RQRR-CMK also reacts with other serine proteases, as prostasin could be detected in the streptavidin pull-downs of biotin-RQRR-CMK labeled cells when analyzed by Western blotting using a prostasin specific antibody (data not shown), emphasizing that specificity of the assay depends on the antibody used for the Western blot analysis.

### Biotin-RQRR-CMK does not react with the matriptase-HAI-1 complex

**Figure 3:**
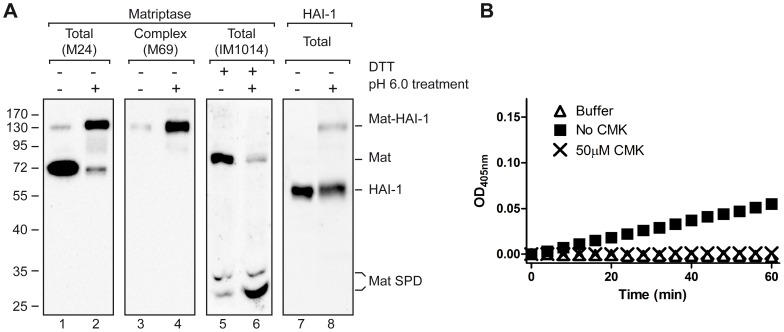
Arg614-cleaved matriptase is able to form complexes with HAI-1 and biotin-RQRR-CMK at pH 6.0. (A) Eleven days post-confluent filter-grown Caco-2 cells were treated with either a physiologically phosphate buffer pH 6.0 for 20 min (lanes 2, 4, 6, and 8) from both the apical and the basolateral side or left untreated (lanes 1, 3, 5, and 7). Cells were lysed and lysates were analyzed by Western blotting using antibodies against total matriptase (M24; lanes 1 and 2), matriptase SPD (IM1014; lanes 5 and 6), matriptase-HAI-1 complex (M69; lanes 3 and 4) and HAI-1 (lanes 7 and 8). Samples in lanes 1–4, 7, and 8 were not boiled to avoid dissociation of matriptase-HAI-1 complexes, while samples in lanes 5 and 6 were boiled and reduced to dissociate the S-S bridged SPD from the stem domain of activated matriptase in order to distinguish between the SEA domain-cleaved form (70 kDa) and the Arg614 cleaved form (25–30 kDa). Treatment with phosphate buffer pH 6.0 and DTT is indicated by +/−. Positions of the molecular weight markers (kDa) are indicated on the left. (B) A solution of 0.2 µM SPD was incubated for 10 min at 37°C with (crosses) or without (squares) 50 µM biotin-RQRR-CMK before addition the chromogenic substrate to a final concentration of 300 µM. All experiments were performed in 20 mM citric acid buffer pH 6.0, 140 mM NaCl and 0.1% BSA at 37°C. Results shown are representative of 3 independent experiments.

After Arg614 cleavage, matriptase rapidly forms a non-covalent, but SDS-resistant complex with HAI-1 that can be detected by SDS-PAGE under non-reducing conditions [32]. In order to investigate whether biotin-RQRR-CMK binds to the matriptase-HAI-1 complex, we took advantage of the fact that cells exposed to slightly acidic conditions have been reported to rapidly convert matriptase from the SEA domain-cleaved form into the Arg614-cleaved form of matriptase in complex with HAI-1 [21]–[23]. First, we verified that exposure to slightly acidic conditions also converts SEA domain-cleaved matriptase into a complex of Arg614-cleaved matriptase with HAI-1 in Caco-2 cells. For this purpose, 11 days post-confluent filter-grown Caco-2 cells were treated with physiological phosphate buffer pH 6.0 for 20 min (figure 3A, lanes 2, 4, 6, and 8) or left untreated (figure 3A, lanes 1, 3, 5, and 7). The cells were lysed and the two lysates were investigated by Western blotting under non-boiled and non-reducing conditions (figure 3A, lanes 1–4 and 7–8) and under reducing conditions (figure 3A, lanes 5 and 6) using different antibodies recognizing matriptase, HAI-1, and their complex. Untreated Caco-2 cells mainly contained matriptase that is not in complex with HAI-1 as it migrates as a 70 kDa band under non-boiled and non-reducing conditions (figure 3A, lane 1). This material is mainly in the SEA domain-cleaved form, as it migrates at 85 kDa under reducing conditions (figure 3A lane 5). Caco-2 cells treated with a pH 6.0 buffer contain matriptase mainly in complex with HAI-1, as it migrates as 130 kDa under non-boiled and non-reducing conditions (figure 3A, lanes 2 and 4) and can be detected using antibodies against HAI-1 (figure 3A, lane 8). Most of this material is in the Arg614-cleaved form, as it migrates as two bands around 25–30 kDa under reducing conditions (figure 3A, lane 6). Thus, treatment with physiological phosphate buffer pH 6.0 induces cleavage of matriptase after Arg614 and complex formation with HAI-1 in Caco-2 cells.

**Figure 4:**
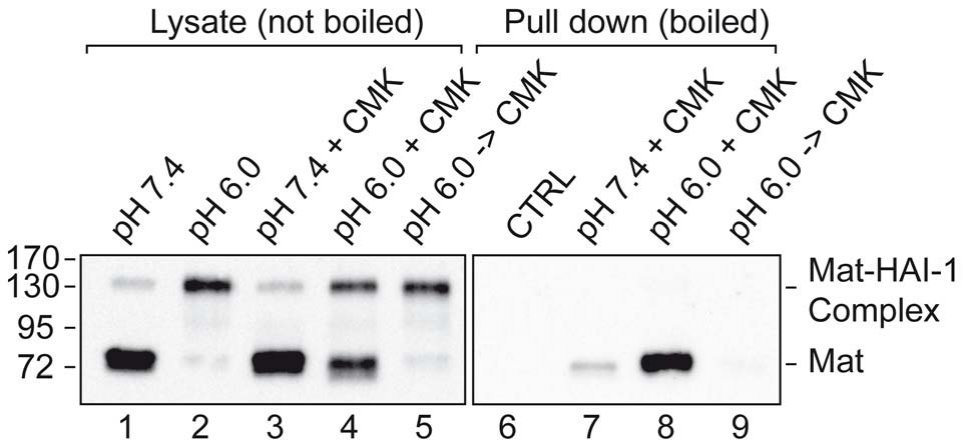
Biotin-RQRR-CMK does not react with matriptase-HAI-1 complexes. Eleven days post-confluent Caco-2 cells grown on Transwell filters were labeled with 50 µM biotin-RQRR-CMK at pH 7.4 (lanes 3 and 7), in physiological phosphate buffer pH 6.0 (lanes 4 and 8), or at pH 7.4 with a 30 min pre-incubation treatment with physiological phosphate buffer pH 6.0 (lanes 5 and 9) for 30 min at 37°C. All cells were lysed and samples of lysates were analyzed under non-boiled and non-reducing conditions (lanes 1–5). Labeled proteases in the lysates were precipitated using streptavidin-coated resin and released from the beads by boiling (lanes 7–9). The streptavidin pull downs were analyzed by SDS-PAGE and Western blotting (lanes 6–9). As a negative control, lysate of cells treated with only physiological phosphate buffer pH 6.0 for 30 min was also streptavidin-precipitated and analyzed (CTRL, lanes 6). All lanes were analyzed using the M32 antibody. Positions of the molecular weight markers (kDa) are indicated on the left. Results shown are representative of 3 independent experiments.

To investigate whether Arg614-cleaved matriptase is able to react with biotin-RQRR-CMK at pH 6.0, we measured the catalytic activity of matriptase SPD at this pH in the presence and absence of biotin-RQRR-CMK in the chromogenic assay described above (figure 1). Matriptase SPD was able to cleave the chromogenic substrate at pH 6.0, although at a lower rate than at neutral pH (compare figure 3B, squares to figure 1B, squares). When 50 µM biotin-RQRR-CMK was added to the reaction, no cleavage of the substrate was observed (figure 3B, crosses). Thus, biotin-RQRR-CMK is still able to inhibit matriptase SPD-mediated substrate hydrolysis at pH 6.0, which allows us to test whether biotin-RQRR-CMK can label matriptase in complex with HAI-1. For this purpose, we employed physiological phosphate buffer pH 6.0 to induce cleavage of matriptase after Arg614 and matriptase-HAI-1 complex formation. Eleven days post-confluent filter-grown Caco-2 cells were treated with 50 µM biotin-RQRR-CMK from the basolateral side at 37°C for 30 min under different conditions; labeling with biotin-RQRR-CMK at pH 7.4 (figure 4, lanes 3 and 7), labeling with biotin-RQRR-CMK in physiological phosphate buffer pH 6.0 (figure 4, lanes 4 and 8), or pre-treatment with physiological phosphate buffer pH 6.0 for 30 min followed by labeling with biotin-RQRR-CMK at pH 7.4 for 30 min (figure 4, lanes 5 and 9). Additional controls were lysates of untreated cells (figure 4, lane 1) and cells treated with physiological phosphate buffer pH 6.0 (figure 4, lane 2) to confirm pH 6.0 induced complex formation between matriptase and HAI-1. In all cases, aliquots of the total lysates (figure 4, lanes 1–5) as well as the boiled streptavidin pull downs were analyzed in Western blot analysis (figure 4, lanes 6–9).

Exposure to low pH clearly induced matriptase-HAI-1 complex formation (figure 4, compare lanes 1 and 2). Biotin-RQRR-CMK labeling of matriptase was detected when labeling was performed at pH 7.4 (figure 4, lane 7) and pH 6.0 (figure 4, lane 8) but not when the cells had been exposed to pH 6.0 for 30 min to induce matriptase-HAI-1 complex formation before labeling with biotin-RQRR-CMK figure 4, lanes 9). These results suggest that matriptase-HAI-1 complex is not labeled with biotin-RQRR-CMK. It was also observed that HAI-1 and biotin-RQRR-CMK compete for binding to matriptase as reduced levels of matriptase-HAI-1 complex is formed in the presence of biotin-RQRR-CMK as compared to in the absence of biotin-RQRR-CMK (figure 4, compare lanes 2 and 4). Higher levels of biotin-RQRR-CMK reactive matriptase was observed when labeling was performed at pH 6.0 as compared to pH 7.4 (figure 4, compare lanes 7 and 8) despite the lower specific activity of matriptase at pH 6.0 compared to pH 7.4 (figure 1B squares compared to figure 3B squares and [33]). Presumably cleavage of matriptase after Arg614 occurs both at pH 6.0 and at pH 7.4 albeit not at the same rate. We interpret the increased formation of biotin-RQRR-CMK labeling at pH 6.0 to be due to a time window between cleavage of matriptase after Arg614 and formation of the matriptase-HAI-1 complex.

### Biotin-RQRR-CMK reacts with both SEA domain-cleaved and Arg614-cleaved matriptase

**Figure 5:**
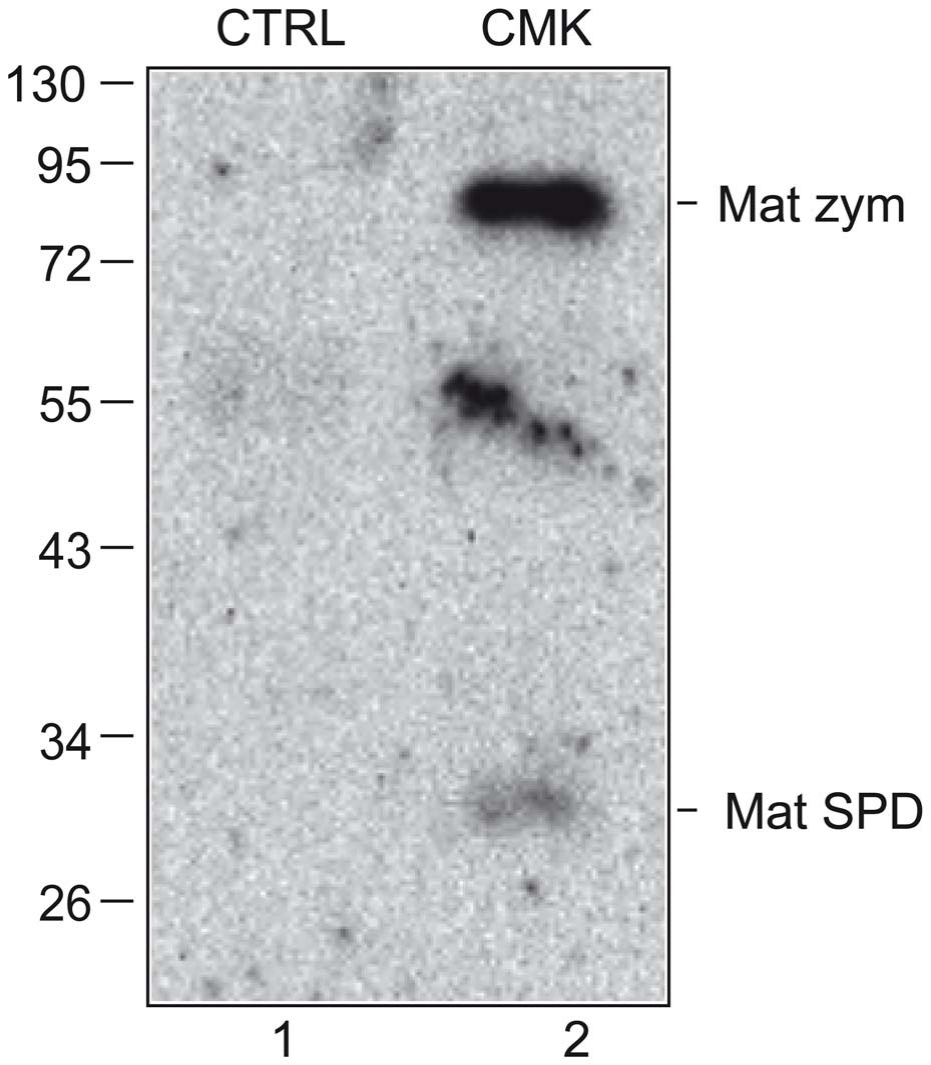
Biotin-RQRR-CMK detects both SEA domain-cleaved zymogen matriptase and Arg614-cleaved matriptase. Eleven days post-confluent Caco-2 cells grown on Transwell filters were labeled with 50 µM biotin-RQRR-CMK from the basolateral side for 180 min at 37°C. As a negative control, cells were labeled from the basolateral side with 50 µM control peptide; biotin-RQRR (CTRL), under the same conditions. Labeled proteases were precipitated using streptavidin-coated resin and the streptavidin pull downs were analyzed by reducing SDS-PAGE and Western blotting using the IM1014 antibody raised against matriptase SPD. Positions of the molecular weight markers (kDa) are indicated on the left and position of SEA domain-cleaved zymogen matriptase and matriptase SPD is indicated on the right. Results shown are representative of 3 independent experiments.

To investigate whether the biotin-RQRR-CMK reactive matriptase present on Caco-2 cells includes both SEA domain-cleaved and Arg614-cleaved matriptase, 11 days post-confluent filter-grown Caco-2 cells were labeled with biotin-RQRR-CMK from the basolateral side for 180 min at 37°C. Biotin-RQRR-CMK labeled proteases were precipitated with streptavidin-coated resin, and subjected to reducing SDS-PAGE. Samples were analyzed by Western blot analysis using an antibody against matriptase SPD (IM1014). Under reducing conditions, matriptase could be detected both as the 70 kDa form representing SEA domain-cleaved matriptase and the 25-30 kDa forms representing Arg614-cleaved matriptase (figure 5, lane 2). No matriptase could be detected when labeling Caco-2 cells with the control peptide; biotin-RQRR (figure 5, lane 1: CTRL). This indicates that both Arg614-cleaved matriptase not in complex with HAI-1 and the SEA domain-cleaved matriptase zymogen is present on the basolateral plasma membrane of Caco-2 cells.

### Biotin-RQRR-CMK also reacts with murine matriptase

**Figure 6:**
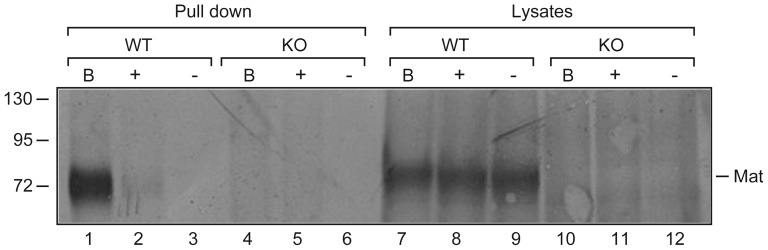
Detection of active matriptase in cultured primary murine keratinocytes. Murine keratinocytes were isolated from newborn wildtype (WT) or matriptase-deficient pups (KO) and cultured on collagen-coated plastic. The cells were grown until sub-confluent and then labeled with S-NHS-SS-biotin (lanes 1, 4, 7, and 10), or with 50 µM biotin-RQRR-CMK (lanes 2, 5, 8, and 11), or with 50 µM control peptide; biotin-RQRR (lanes 3, 6, 9, and 12). Cells were lysed and analyzed on Western blot (lanes 7–12). The remaining lysates were precipitated using streptavidin-coated resin, released from the beads by boiling under non-reducing conditions and lysates were analyzed by SDS-PAGE and Western blotting using the matriptase antibody AF3946. Results shown are representative of 2 independent experiments.

The described assay can be easily modified to detect active matriptase from other species, as the specificity of the assay depends only on the antibody used. To verify this, the presence of active matriptase was investigated in cultured primary murine keratinocytes. Keratinocytes from the skin of matriptase wildtype (WT) and matriptase-deficient (KO) newborn pups were isolated, plated on collagen-coated plastic and labeled with 50 µM biotin-RQRR-CMK at subconfluence for 180 min at 37°C or labeled with S-NHS-SS-biotin for 30 min at 4°C to assess surface-associated matriptase. As a negative control, keratinocytes were labeled with biotin-RQRR. Equal aliquots of cleared lysates were analyzed by Western blot for total matriptase, prior to streptavidin pull down of biotinylated proteins. Proteins were released from the streptavidin-coated resin by boiling in SDS sample buffer and analyzed by Western blotting. While at very low levels, we were able to detect biotin-RQRR-CMK reactive matriptase by labeling of keratinocytes isolated from mice expressing matriptase with biotin-RQRR-CMK (figure 6, lane 2). No matriptase was detected when labeling with the corresponding control peptide; biotin-RQRR (figure 6, lane 3). No matriptase could be detected in lysates or pull downs of keratinocytes from matriptase-deficient mice (figure 6, lanes 4–6 and 10–12), whereas matriptase was easily detected in all lysates of the 3 differently treated keratinocyte cultures from mice expressing WT matriptase (figure 6, lanes 7–9). Comparison of S-NHS-SS biotin labeling and biotin-RQRR-CMK labeling of WT murine keratinocytes showed that only a fraction of surface-associated matriptase on WT keratinocytes cells could be detected by means of biotin-RQRR-CMK (figure 6, compare lanes 1 and 2). This suggests that biotin-RQRR-CMK and the described assay can be applied for a range of cell systems as well as other species when an appropriate antibody is used.

## Discussion

Due to the importance of the membrane-anchored serine protease matriptase in physiological and pathological processes [2]–[4], [38]–[43], it would be advantageous to be able to detect active matriptase in cell culture samples in a simple and inexpensive way. Several assays to detect active matriptase have been established, but they are either indirect [18], [36] or technical demanding, e.g. requiring access to a 2D fluorescent imager [44].

This study describes a simple and efficient assay for detection of active matriptase in cultured cells by combining antibody specificity, the high affinity of the biotin-streptavidin interaction, and a specifically designed chloromethyl ketone peptide inhibitor; biotin-RQRR-CMK.

In the present study, live cells in culture were allowed to react with biotin-RQRR-CMK for a period of time after which surplus biotin-RQRR-CMK was thoroughly washed away, followed by lysis of the cells and isolation and analysis of the biotinylated proteins. It is therefore unlikely, that activation of matriptase and/or labeling occurs after lysis of the cells. We have used Caco-2 cells as a model in the present study since Caco-2 cells have an endogenous expression of matriptase and all necessary auxiliary proteins required for matriptase activation. We have previously shown that cleavage of matriptase after Arg614 takes place on the basolateral plasma membrane of 11 days post confluent Caco-2 cells [36], confirming that matriptase is activated in these cells.

Recent studies show that the SEA domain-cleaved zymogen form of matriptase is at least partially biologically active [13], [14], [45]. It is accepted that the zymogen form of serine proteases can exhibit proteolytic activity without being proteolytically activated. Zymogen activity is described by the zymogenicity factor, which is the ratio between the catalytic efficiency of the activated form and the zymogen form. The zymogenicity for most proteases is in the range of 10^3^–10^6^, which means that the zymogen is virtually inactive [46]. However, for some proteases the zymogenicity is as low as 3–9 as seen for tPA [47]–[51]. The zymogenicity for rat matriptase towards a small model substrate *in vitro* has been determined to 27 [13], [14], [52]. This suggests that the zymogen form of matriptase has significant proteolytic acitivity. It is generally believed that zymogens as in the case of matriptase alternates between different conformations, one in which the conformation of the active site and underlying catalytic machinery resemble that of the active enzyme [51] and it can therefore be expected that only part of the SEA-domain cleaved zymogen matriptase is in the active conformation.

We would ideally prefer the assay to detect the Arg614-cleaved form of matriptase in addition to the active SEA domain-cleaved zymogen form, but not the inactive SEA domain-cleaved form and not the inhibitor-bound form of matriptase.

We clearly show that biotin-RQRR-CMK reacts with the Arg614-cleaved form of matriptase, as represented by both purified SPD and matriptase endogenously expressed by Caco-2 cells. Biotin-RQRR-CMK inhibits matriptase SPD-mediated substrate hydrolysis and biotin-RQRR-CMK-labeled matriptase can be detected as 25–30 kDa bands under boiling and reducing conditions from lysates of 11-days post confluent Caco-2 cells as would be expected of Arg614-cleaved matriptase (figure 5, lane 2).

We have previously shown that at steady state, matriptase expressed by 11 days post confluent Caco-2 cells is primarily present on the plasma membrane [36] and mainly present in the SEA domain-cleaved zymogen form (figure 3, lane 5). Our results show that biotin-RQRR-CMK reacts with the SEA domain-cleaved matriptase, as labeling of Caco-2 cells with biotin-RQRR-CMK also result in a 85 kDa band under boiled and reducing conditions as would be expected for the active SEA domain-cleaved matriptase.

Biotin-RQRR-CMK is an efficient inhibitor of matriptase at the concentrations used and biotin-RQRR-CMK and NHS-SS-biotin have approximately the same molecular weight and half-life. However, even though we detected an efficient labeling of matriptase on the basolateral plasma membrane with NHS-SS-biotin, only low levels of biotin-RQRR-CMK-reactive matriptase could be detected. This suggests that large amount of SEA domain-cleaved matriptase was present and accessible but only a fraction of it in a biotin-RQRR-CMK reactive form. Our interpretation of this is that biotin-RQRR-CMK only reacts with the “active” form SEA domain-cleaved zymogen form of matriptase, which only constitute a fraction of the total SEA domain-cleaved matriptase.

The biotin-RQRR-CMK seems not to react with matriptase in complex with HAI-1. We show that SEA domain-cleaved matriptase after exposure to pH 6.0 for 30 min at 37°C efficiently induces activation of matriptase and matriptase-HAI-1 complex formation. We have previously shown that endocytosis of biotinylated matriptase-HAI-1 complex from the basolateral plasma membrane of 11 days post-confluent Caco-2 cells takes more than an hour [36]. In the present study, a 30 min incubation at pH 6.0 was needed for maximum matriptase-HAI-1 complex formation to take place which was followed by treatment with biotin-RQRR-CMK. We therefore expect that at least part of the matriptase-HAI-1 complex formed after exposure to pH. 6.0 for 30 min were exposed to biotin-RQRR-CMK suggesting that matriptase-HAI-1 does not bind the biotin-RQRR-CMK inhibitor.

Thus the data suggest that the biotin-RQRR-CMK peptide reacts with two active forms of matriptase; the Arg614-cleaved form and the active SEA domain-cleaved form but not the inactive SEA domain uncleaved form and not with the matriptase-HAI-1 complex.

For most proteases, the serine protease domain cleaved form is also the biologically active form. However, we recently showed that zymogen-locked matriptase is able to activate prostasin [45]. This suggests that at least under some circumstances the SEA domain-cleaved zymogen form of matriptase is the biologically active form. It has been difficult experimentally to detect the non-complexed Arg614-cleaved active form of matriptase [21], [53]. This is probably due to a low abundance of non-complexed Arg614-cleaved active matriptase. However, data obtained in the present study suggests that Arg614-cleaved matriptase not in complex with HAI-1 does exist albeit at very low concentrations as biotin-RQRR-CMK clearly binds Arg614-cleaved matriptase. For some time it was speculated that the matriptase-HAI-1 complex was formed before or concomitantly with matriptase cleavage after Arg614. However, when comparing biotin-RQRR-CMK labeling at pH 7.4 with labeling at pH 6.0 it is clear that even though matriptase activity is lower at pH 6.0 (figure 3B squares, [33]) than at pH 7.4 (figure 1B squares) more matriptase reacts with biotin-RQRR-CMK at pH 6.0. This suggests that a time window exists, in which reaction with biotin-RQRR-CMK can take place, subsequent to matriptase cleavage after Arg614 and before the matriptase-HAI-1 complex is formed.

We found that HAI-1 and biotin-RQRR-CMK compete for binding to matriptase. This corresponds well with the recently published structure of matriptase in complex with HAI-1 showing that Kunitz domain 1 of the protease inhibitor interacts with the substrate binding cleft of matriptase [28]. Furthermore, although HAI-1 binds matriptase in a reversible, but yet very stable manner, biotin-RQRR-CMK is not able to dissociate the matriptase-HAI-1 complex under the conditions used in the study although biotin-RQRR-CMK is present at high concentrations.

The presence of Arg614 cleaved matriptase as detected by analysis of total cell lysates by western blot under reducing conditions using the IM1014 antibody indicates the amount of Arg614 cleaved matriptase present in the sample, but not whether the Arg614 cleaved matriptase is unbound and active or if matriptase is bound to HAI-1 and therefore inactive. Actually in some cases low amount of Arg614 cleaved matriptase can be detected (figure 3, lane 5) of which some reacts with biotin-RQRR-CMK indicating that active matriptase is present (figure 4, lane 7). Whereas the same cells after exposure to pH 6.0 contains larger amounts of Arg614 cleaved matriptase (figure 3, lane 6) of which no biotin-RQRR-CMK reactive matriptase can be detected (figure 4, lane 9) indicating the absence of active matriptase. In contrast to analysis of the total lysate using IM1014 on Western blots, the biotin-RQRR-CMK assay offers the possibility to detect (and distinguish between) the active SEA domain-cleaved zymogen form and the non-complexed Arg614-cleaved form of matriptase.

In summary, we have established an assay for detection of active matriptase on cells in culture. The availability of a simple and inexpensive assay for detection of active matriptase may help us understand the complex regulation of matriptase activity. The assay described here can be transferred to other species as shown by detection of active matriptase in primary cultures of mouse keratinocytes.
